# Gender differences in the association between marriage and epigenetic age

**DOI:** 10.3389/fragi.2026.1931190

**Published:** 2026-09-07

**Authors:** Theo O. Moran, Alina M. Kushner, Theodore F. Robles, Jennifer W. Robinette

**Affiliations:** 1 Psychology Department, Crean College of Health and Behavioral Sciences, Chapman University, Orange, CA, United States; 2 Department of Psychology, University of California, Los Angeles, CA, United States

**Keywords:** accelerated aging, biological age, epigenetic age, gender, marital status

## Abstract

**Introduction:**

Being married is related to better health, but many studies reveal greater health benefits among men relative to women. Some research has indicated that married individuals exhibit decelerated epigenetic age relative to individuals who are not married, but questions remain regarding gender differences therein. The purpose was to investigate gender differences in associations of marital status to epigenetic age (ing) assessed on three clocks/algorithms (PhenoAge, GrimAge, and DunedinPACE).

**Methods:**

Data from 762 participants in the national Midlife in the United States Study (MIDUS II; men *N* = 382, women *N* = 380) were used. Weighted linear regressions were conducted to test the hypotheses that 1) being married would be associated with decelerated epigenetic age (ing) and that 2) this association would be stronger among men than women.

**Results:**

Being married, relative to being separated or divorced, was associated with decelerated epigenetic aging on DunedinPACE, although not PhenoAge or GrimAge. Investigating gender differences revealed a divergence regarding never marrying, a marital status that accelerated GrimAge and DunedinPACE scores among men, but not women.

**Discussion:**

These results contribute new knowledge about marital status and early risk for disease, as indicated by accelerated pace of aging among men and women whose relationships dissolved. More research is needed to identify factors explaining why men who never marry may be at risk for accelerated epigenetic aging while women are not.

## 1 Introduction

“To get the full value of joy, you must have someone to divide it with” (Mark Twain). As this quote suggested, much of the joy people experience comes from meaningful social relationships. Perhaps one of the most central relationships in an adult’s life is that with one’s spouse. Beyond joy, a wealth of research has demonstrated that marriage coincides with good health, with married individuals having a lower risk for outcomes such as stroke and cancer- and cardiovascular-related mortality than their never married, separated, divorced, and widowed counterparts (Dupre, 2016; Rendall et al., 2011; Roelfs et al., 2012; Wang Y. et al., 2020; Zueras et al., 2020). Many of these inquiries have also revealed, however, that the circumstance of being married is differentially associated with health by gender often preferentially benefitting the health of men over that of women (Rendall et al., 2011; Zueras et al., 2020).

Unique patterns of DNA methylation (DNAm) quantified in what has been referred to as epigenetic “clocks” or “algorithms” are related to premature aging which, in turn, sets the trajectory for multiple morbidities and mortality (Kusters and Horvath, 2025; Seale et al., 2022). It follows to ask, then, whether the relatively enhanced health status of married individuals extends to decelerated age or aging on such epigenetic clocks/algorithms. Somewhat recent research has indicated that married individuals indeed exhibit younger epigenetic age, or smaller discrepancies between their chronological and biological age, when compared to unmarried individuals in large national samples of midlife and older adults (Prigerson et al., 2025; Rentscher et al., 2023). Given the utility of these epigenetic clocks in characterizing individuals early in the disease process (Kusters and Horvath, 2025; Seale et al., 2022), examining epigenetic age may shed further light on health disparities that stem, at least in part, by marital circumstances. The present study investigated epigenetic age on two “second-generation” clocks, i.e., PhenoAge and GrimAge, and one pace of aging algorithm, DunedinPACE (Belsky et al., 2022; Levine et al., 2018; Lu et al., 2019), in the context of marital status and gender, and critically, interactions between the two. The selection of these clocks was based on recent research suggesting that these second-generation clocks and algorithms are more sensitive so social determinants of health such as socioeconomic status and race/ethnicity than are first-generation clocks such as Hannum and Horvath which were trained on chronological age (Cuevas et al., 2024; Markon et al., 2024; Willems et al., 2025; although see Supplementary Table S1). Extrapolating from that research, the current study investigated marital status as another social status for its association with epigenetic aging. If the gender differences in observed associations between marital status and health extend to these methylation-based markers that associate with risk for morbidity and mortality, the present research would not only add new knowledge to the marital status and health literature but would also encourage more discussion about features of marriage that may not confer the same benefits, or may even contribute to more strain, between men and women.

### 1.1 Marital status and health

Nearly half a century ago, researchers documented that married individuals had fewer acute and chronic health conditions and better physical functioning than widowed individuals who, in turn, had better health than separated and divorced individuals (Verbrugge, 1979). Investigations into the historical stability of these associations over long periods of time (nearly 50 years) have revealed a somewhat unchanging pattern, whereby separated, divorced, and widowed individuals reported worse health than their married counterparts (Liu and Umberson, 2008). More recent studies have demonstrated that married individuals were less physically frail, had fewer problems with activities of daily living, and had lower mortality risk in cross-sectional and longitudinally designed studies (Holt-Lunstad et al., 2008; Jia and Lubetkin, 2020; Kojima et al., 2020; Manvelian and Sbarra, 2020; Rendall et al., 2011). Some research suggests that a reason for this association between marriage and better health outcomes is the early detection of diseases such as cancer (Chen Z. et al., 2021).

Despite this evidence indicating the consistent benefits of marriage for physical health outcomes, several investigations have revealed gender differences in these benefits (Umberson and Thomeer, 2020). Married men have lower risk for cancer-, cardiovascular-, and all-cause mortality than their unmarried counterparts, and although married women have lower mortality risk than unmarried women, this differential benefit from marriage is often attenuated among women compared to men (Aizer et al., 2013; Rendall et al., 2011; Wang Y. et al., 2020). Some evidence suggests that the specific unmarried status that poses the greatest risk for mortality depends on gender as well (Mohammad Hashem et al., 2025). At least one study demonstrated that, over a 6-year period, men who remained single or became widowers were more likely to develop frailty than married men (Trevisan et al., 2020). In this same study, however, there was no difference in frailty risk between marital groups among women (but see Trevisan et al., 2020). Another longitudinal study found that men who were consistent widowers reported significantly worse health than men who were consistently married, but this difference was not observed among women (Williams and Umberson, 2004). Not only do the above studies demonstrate that marriage is sometimes more health-salutary for men, but some evidence has indicated that marriage may at times be related to health problems for women. In one study, being married or partnered was a risk factor for hypertension among women, but not men (Tuoyire and Ayetey, 2018). These studies demonstrated that being married was more beneficial, and becoming widowed was more detrimental, for men than women (but see Brenn and Ytterstad, 2016).

While these studies suggested a greater health benefit from marriage among men, there are sometimes findings demonstrating the opposite pattern or no gender difference. Wang L. et al. (2011), for example, found no gender difference in associations between marital status and colon cancer mortality, although survival after diagnosis with local or regional stage colon cancer was increased via marriage more so for women than men. Similarly, in a meta-analysis examining survival from multiple cancer types, Krajc et al. (2023) found few gender differences in the benefits of being married. The one exception was that being married, relative to being divorced/separated, increased survival from specific types of cancer more for men than women. One other study found that, although risk for mortality after losing a spouse was attenuated at older ages, this attenuated risk was only observed among men, not women (Brenn and Ytterstad, 2016). Other researchers reported no gender differences for marriage with respect to stroke incidence (Dupre, 2016), cardiovascular mortality (Schulz and Beach, 1999; Wong et al., 2018), and all-cause mortality (Manzoli et al., 2007).

One reason for the inconsistent patterns of gender differences in the marriage-health literature may be due to selection; the benefits of marriage attenuate with age, particularly for men, likely reflecting selection effects whereby greater baseline resiliency renders any further marital benefits for men as marginal (Franke and Kulu, 2017; Manzoli et al., 2007; Staehelin et al., 2012). Many of the studies reporting no gender differences in associations between marriage and health were conducted with samples of older adults (Dupre, 2016; Manzoli et al., 2007; Schulz and Beach, 1999). Additionally, a notable caveat to most published work in this area is an over-reliance on analytic approaches that stratify analyses by gender, rather than testing for gender differences formally (Manzoli et al., 2007; Wang L. et al., 2011). Multiplicative interaction terms between marital status and gender are required to identify statistically significant differences in marriage-health (Cotter et al., 2023; Gelman and Stern, 2006).

### 1.2 Epigenetic age and health

The health complications discussed thus far, and indeed mortality, have underlying biological mechanisms (Kusters and Horvath, 2025; Moosavi and Motevalizadeh Ardekani, 2016; Seale et al., 2022). In fact, age is among the strongest risk factors for morbidity and mortality (Niccoli and Partridge, 2012). Prior research has linked marriage to established biological markers of aging such as telomere length (Chen R. et al., 2020; Mainous et al., 2011; Whisman et al., 2016; Yen and Lung, 2013). DNA replication over the lifespan progressively shortens telomeres, but the rate of telomere shortening varies by person and, as such, is used to assess age acceleration (Blackburn et al., 2015; Shammas, 2011). Married individuals tend to display longer telomeres than their unmarried counterparts, consistent with a comparatively younger biological age (Chen R. et al., 2020; Mainous et al., 2011; Whisman et al., 2016; Yen and Lung, 2013). These findings suggest that marital status is biologically embedded and provides at least one plausible pathway linking marital status to health outcomes and mortality risk.

Epigenetic aging, or life course patterns of methylation along the human genome, is a distinct form of biological aging (Jylhävä et al., 2017; Kabacik et al., 2022; Seale et al., 2022). Age acceleration, or greater discrepancies between chronological and epigenetic age, is related to a host of negative outcomes including cognitive and physical dysfunction, kidney dysfunction, chronic health conditions, and mortality (Chen B. H. et al., 2016; Faul et al., 2023; Jylhävä et al., 2017; Surachman et al., 2026). The health complications associated with accelerated epigenetic age are observed at greater prevalence among unmarried individuals compared to married individuals (Dupre, 2016; Rendall et al., 2011; Roelfs et al., 2012; Wang Y. et al., 2020; Zueras et al., 2020). Recent research has observed accelerated epigenetic age assessed on clocks such as PhenoAge and GrimAge, and accelerated pace of aging on epigenetic algorithms like DunedinPACE, among samples of older adults without a marital partner compared to those with a partner (Prigerson et al., 2025; Rentscher et al., 2023).

At least four studies have examined gender differences in marital status-epigenetic aging associations (Cui et al., 2025; Wang J. et al., 2025; Wang W. et al., 2023; Yu, 2023). All but one reported that married individuals had relatively decelerated epigenetic age when compared to separated, divorced, and widowed individuals; Wang J. et al. (2025) found that never married individuals had greater risk for accelerated epigenetic age, while divorced, separated, and widowed individuals had decreased risk compared to married adults. Gender differences observed in these reports are inconsistent and, like many marital status-health studies, most resulted from analyses stratified by gender. While sufficient for exploratory analyses, it is difficult to draw conclusions from stratified models that provide no formal statistical test for the significance of potential gender differences (Cotter et al., 2023; Gelman and Stern, 2006). Only Wang W. et al. (2023) used multiplicative interactions between gender and marital status. In that study, however, specific coefficients of the multiplicative interactions were not reported but rather were mentioned to justify reporting and interpreting only the models stratified by gender. The analytic strategies used in the above reports limited the ability to identify which marital category poses the greatest risk for epigenetic age or aging, and whether that, too, depends on gender.

### 1.3 The present study

To evaluate epigenetic aging, this study utilized three advanced algorithms: GrimAge, PhenoAge, and DunedinPACE. These second- and third-generation tools were trained on clinical biomarkers for disease and measure epigenetic age acceleration, providing the most accurate indicators currently available for predicting healthspan, overall mortality risk, and the development of chronic diseases (Belsky et al., 2022; Levine et al., 2018; Lu et al., 2019). The present study builds on existing literature in two ways. First, through interacting gender with multiple specific marital categories, the present analyses will formally investigate the statistical significance of gender differences in the experience of each marital status category. Second, the present study utilizes data from the Midlife in the United States survey (MIDUS II) which represents a wide age range of U.S. adults. The first hypothesis in the present study was that being separated/divorced or never married, relative to being married, would relate to accelerated age on the PhenoAge and GrimAge clocks, and accelerated pace of aging on the DunedinPACE algorithm. The second hypothesis was that the above associations would be stronger among men than women. MIDUS II is a national sample of U.S. men and women with ages spanning from midlife to older adulthood and was initiated out of the observation that much research investigating developmental origins of disease focus on childhood and research on aging focuses on older adulthood, with much less attention spent on unique risks in midlife. Findings from the present study have the potential to reveal gender-specific early risk for disease that may stem, in part, from marriage and extend findings in other research exclusively focusing on older adults (Rentscher et al., 2023) or adults from other nations (Wang W. et al., 2023).

## 2 Materials and methods

### 2.1 Participants and procedure

The present study used data from the second wave of the MIDUS. Begun in 1994, MIDUS is an ongoing national survey examining the health of men and women in the U.S aged 25–74 years. Participants were recruited via random digit-dialing procedures and specific city oversamples, and although not included in the present analysis, siblings and twins of participants in the random sample were also recruited, as were an oversample of African Americans living in the Milwaukee, WI. The core survey was conducted by telephone interview and self-administered questionnaire and investigated participants’ sociodemographic backgrounds and health including several questions about participants’ relationships (Ryff et al., 2017a). A second wave (MIDUS II) was conducted in 2004 and included sub-studies including the Biomarker Study. Participants in the Biomarker Study stayed overnight in one of three Clinical Research Centers across the U.S during which time blood samples were collected from participants (Ryff et al., 2019). In 2011, a Refresher sample was recruited to replenish the original MIDUS cohort that involved the same data collection efforts (Ryff et al., 2017b; Weinstein et al., 2019). The present study combined data from MIDUS II and the Refresher. In 2022, the Genomic Study was launched to extract DNA methylation data (DNAm) from 1,310 Biomarker Study blood samples collected from the MIDUS II and Refresher cohorts and construct a series of epigenetic clocks and algorithms, the primary outcomes in the present study (University of Wisconsin Institute on Aging and University of California Los Angeles Social Genomics Core Laboratory, 2023). The present analysis did not include siblings, twins, or Milwaukee oversampled adults, leaving 833 participants with data on the epigenetic outcomes. One participant was missing data education, another three were missing responses to the question asking about marital status, 11 were missing data on race/ethnicity, and 25 were missing data on body mass index. Of these participants, only 31 had been widowed, and given this small sample size, widows were dropped from the current analysis, leaving an analytic sample of *n* = 762. MIDUS staff constructed post-stratification weight variables for the randomly digit-dialed participants used in the current analysis that adjusted for differences between the original MIDUS and Refresher samples and the U.S. population using the 1995 and 2012 Census Current Population Surveys, respectively (Palit et al., 2016). All participants signed consent forms before participating in MIDUS projects. Additionally, all data collection procedures were approved by the University of Wisconsin, Madison Ethical Review Board.

### 2.2 Measures

#### 2.2.1 Epigenetic outcomes

Epigenetic age as assessed on the PhenoAge and GrimAge clocks (Levine et al., 2018; Lu et al., 2019) and pace of epigenetic aging on the DunedinPACE algorithm served as the primary outcomes of the present study (Belsky et al., 2022). The PhenoAge clock was constructed by first developing a measure of phenotypic (i.e., biological) age using chronological age and 9 clinical biomarkers related to aging. Next, an elastic net regression was conducted to identify 513 CpG sites that collectively predict this measure of phenotypic age, enabling an estimation of a phenotypic age from DNAm data (Levine et al., 2018). The GrimAge clock was also constructed in a multi-step process. First, an elastic net regression was used to predict 88 key plasma protein levels as well as smoking pack years by DNAm across all available CpG sites. Next, the DNAm surrogates related to each plasma protein and smoking pack years, chronological age, and gender were regressed with time-to-death due to all-cause mortality in a net cox regression model. This prediction was then translated to a biological age estimate (Lu et al., 2019). The DunedinPACE algorithm indicates an individual’s pace of aging rather than their biological age. It was created by tracking 19 multi-organ system indicators over 20 years and developing an associated pace of aging measure. An elastic net regression was conducted to identify 173 CpG sites that predicted the 20-year pace of aging score (Belsky et al., 2022). Together, these three epigenetic scores provide complementary indications of biological aging. First generation clocks such as Hannum and Horvath, although available in the MIDUS cohorts, were trained on chronological age rather than biomarkers of disease (Hannum et al., 2013; Horvath, 2013). As such, these first-generation clocks were not evaluated in the current study with a focus on marital status, health and aging, and gender differences in these links.

#### 2.2.2 Marital status

Marital status was attained from responses to a single item, “Are you married, separated, divorced, widowed, or never married?” Responses were coded as 1 (*married*), 2 = (*separated or divorced*), and 3 = (*never married*).

#### 2.2.3 Gender

Gender was coded as 0 = *men* and 1 = *women*.

#### 2.2.4 Covariates

Several covariates were included given their known associations with health and aging. For the present study, age was coded in years. The MIDUS II educational attainment variable ranged from 1 = *no school/some grade school* to 12 = *professional degree*, but to collapse categories with small sample sizes, a dichotomous education variable was constructed for which participants were coded as 0 = *less than a 2-year degree or less* and 1 = 4*-year degree or higher*. Participants’ body mass index was calculated by dividing weight in kilograms by height in meters squared. The MIDUS II questionnaire included several items about smoking behaviors including, “Have you ever smoked cigarettes regularly” (“*yes*”/“*no*”) and “Do you smoke cigarettes regularly now” (“*yes*”/“*no*”). Responses to these items were used to construct a smoking variable for which 0 = *never smoked*, 1 = *used to smoke*, and 2 = *currently smoked*. The race/ethnic question available in MIDUS II had codes for *multiracial* (0; *n* = 1), *White* (1; *n* = 628), *Black or African American* (2; *n* = 54), *Native American or Aleutian Islander* (3; *n* = 15), *Asian or Pacific Islander* (4; *n* = 12), and *Other* (5; *n* = 52). However, both the MIDUS II and Refresher samples consisted primarily of non-Hispanic White individuals, and as such, a dichotomous race/ethnicity variable was constructed for which 0 = *non-Hispanic White* participants and 1 = *non-White* participants. To adjust for potential cohort differences in model estimates, an indicator variable was included where 1 = *MIDUS II* and 2 = *Refresher*. Finally, because the number of months that elapsed between the completion of the core interview and the collection of blood samples varied across the study participants, the number of months between these data collection methods was included as a covariate in all analytic models.

### 2.3 Statistical analysis

All analysis was conducted using Stata 18. Comparisons between men and women on all analytic variables were investigated using weighted *t* tests for continuous outcomes and weighted chi^2^ tests for dichotomous (*z* scores) and categorical (chi^2^ scores) variables. Separate weighted linear regressions were conducted to evaluate three epigenetic outcomes. To test the hypothesis that being separated/divorced or never married (relative to being married) would be related to accelerated epigenetic age or pace of aging, Model 1 regressed these epigenetic outcomes on marital status and gender, adjusting for age (10-year increments), educational attainment, race/ethnicity, BMI, smoking, sample (MIDUS II or Refresher), and the number of months between the telephone interview/self-administered questionnaire and the collection of blood samples. To test the hypothesis that the association between marital status and epigenetic age would be stronger among men than women, Model 2 introduced a multiplicative interaction term between marital status and gender.

## 3 Results

### 3.1 Description of analytic sample

Table 1 reports a description of the analytic sample separately by men and women. Of the 762 participants in our analyses, approximately 50 percent were women and 50 percent were men, 70 percent, or 533 participants, were non-Hispanic White participants (30 percent, or 229 participants, were non-White), and 84 percent were in the Refresher cohort (16 percent were from the MIDUS II cohort). Men were exhibiting significantly accelerated epigenetic scores relative to women on PhenoAge (*t =* 2.87 [*df* = 760], *p* < 0.01), GrimAge (*t =* 3.99 [*df* = 759], *p* < 0.001), and DunedinPACE (*t =* 1.97 [*df* = 760], *p* < 0.05). Men were significantly chronologically older than women (*t =* 2.99 [*df* = 760], *p* < 0.01). There were significant gender differences in marital status, with more men married but fewer men divorced/separated or never married than women (*chi* ^*2*^ = 35.66, *p* < 0.001). More men than women were non-Hispanic White (*z* = 3.35, *p* < 0.001). Men and women significantly differed across smoking groups, with more women than men having never smoked, but more men than women who had quit smoking and fewer men than women who reported currently smoking (*chi* ^*2*^ = 10.13, *p* < 0.01). On average, there was one additional month between the completion of MIDUS II telephone interview/self-administered questionnaire and the Biomarker Study among women relative to men (*t = −*2.06 [*df* = 760], *p* < 0.05).

**TABLE 1:** Description of analytic sample, midlife in the United States survey, MIDUS II and Refresher samples.

​	Women (*n* = 380)	Men (*n* = 382)
PhenoAge, *mean (SE)*	41.98 (12.59)	44.83 (14.66)
GrimAge, *mean (SE)*	60.26 (10.02)	63.51 (12.32)
DunedinPACE, *mean (SE)*	0.96 (0.13)	0.98 (0.13)
Married, percent	59.47%	77.49%
Separated/Divorced, percent	24.47%	9.42%
Never married, percent	16.05%	13.09%
Age, *mean (SE)*	50.13 (12.32)	52.95 (13.69)
Two-year degree or less, percent	29.74%	27.75%
Four-year degree or higher, percent	70.26%	72.25%
Non-Hispanic whites, percent	63.95%	75.13%
Racial/ethnic minorities, percent	36.05%	24.87%
MIDUS II core sample, percent	18.42%	14.14%
Refresher, percent	81.58%	85.86%
Body mass index, *mean (SE)*	28.45 (7.89)	28.54 (5.15)
Never smokers, percent	61.58%	59.16%
Smokers who had quit, percent	25.53%	33.51%
Current smokers, percent	12.89%	7.33%
Number of months between projects	24.80 (12.11)	23.13 (10.27)

### 3.2 Epigenetic age, marriage, and gender

Results of the weighted linear regressions predicting three epigenetic outcomes by marital status and gender (Model 1) and their interaction (Model 2) can be found in Table 2. Model 1 evaluated associations between the three epigenetic outcomes and both marital status and gender. Results of Model 1 indicated married participants had significantly decelerated epigenetic scores compared to those who were divorced or separated, but only on the DunedinPACE algorithm (*b =* 0.03*, SE =* 0.01, *p =* 0.02). Men had significantly accelerated epigenetic scores relative to women on the GrimAge (*b =* −1.99*, SE =* 0.31, *p <* 0.001) and DunedinPACE scores (*b =* −0.03*, SE =* 0.01, *p <* 0.001), although there was no significant gender difference on the PhenoAge clock.

**TABLE 2:** Weighted linear regressions predicting three epigenetic outcomes, midlife in the United States survey, MIDUS II and Refresher samples, n = 762.

​	PhenoAge		GrimAge		DunedinPACE	
​	Model 1	Model 2	Model 1	Model 2	Model 1	Model 2
Married						
Separated/Divorced	0.67 (0.63)	0.17 (1.07)	0.47 (0.44)	0.72 (0.74)	0.03*(0.01)	0.01 (0.02)
Never Married	−0.25 (0.64)	0.10 (0.90)	0.81 (0.45)	1.89**(0.63)	0.02 (0.01)	0.05** (0.02)
Women^a^	−0.51 (0.44)	−0.50 (0.54)	−1.99*** (0.31)	−1.55*** (0.38)	−0.03*** (0.01)	−0.02* (0.01)
Married x Gender^a^						
Separated/Divorced	​	0.70 (1.29)	​	−0.56 (0.91)	​	0.02 (0.02)
Never Married	​	−0.63 (1.20)	​	−2.06* (0.84)	​	−0.06** (0.02)
Age (in years)	0.93*** (0.02)	0.93*** (0.02)	0.76*** (0.01)	0.76*** (0.01)	0.00*** (0.00)	0.00*** (0.00)
Four-year degree or higher^b^	0.23 (0.47)	0.23 (0.47)	−0.58 (0.33)	−0.54 (0.33)	−0.01 (0.01)	−0.01 (0.01)
Non-Hispanic White^c^	−0.31 (0.59)	−0.33 (0.59)	−1.71*** (0.41)	−1.79*** (0.41)	−0.05*** (0.01)	−0.05*** (0.01)
Refresher^d^	2.25* (0.91)	2.30* (0.91)	1.68** (0.64)	1.81** (0.64)	0.04* (0.02)	0.04** (0.02)
Body mass index	0.15*** (0.03)	0.15*** (0.03)	0.12*** (0.02)	0.12*** (0.02)	0.01*** (0.00)	0.01*** (0.00)
Never Smokers						
Smokers who had quit	1.11* (0.52)	1.15* (0.53)	2.23*** (0.37)	2.30*** (0.37)	0.03** (0.01)	0.03** (0.01)
Current smokers	2.51*** (0.70)	2.57*** (0.71)	8.42*** (0.49)	8.57*** (0.49)	0.12*** (0.01)	0.13*** (0.01)
Number of months between projects	0.15*** (0.03)	0.15*** (0.03)	0.07*** (0.02)	0.07*** (0.02)	0.00 (0.00)	0.00 (0.00)

All coefficients are b (SE).

^a^ Compared to men.

^b^ Compared to 2-year degree or less.

^c^ Compared to non-White participants.

^d^ Compared to MIDUS II, core sample.

*p < .05. **p < .01. ***p < .001.

**FIGURE 1:**
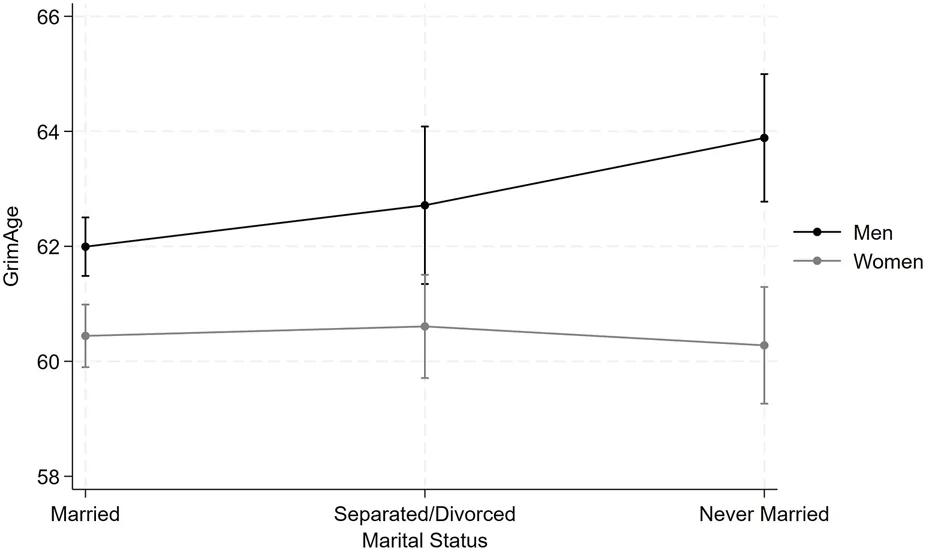
Marital status x gender interaction in relation to GrimAge.

**FIGURE 2:**
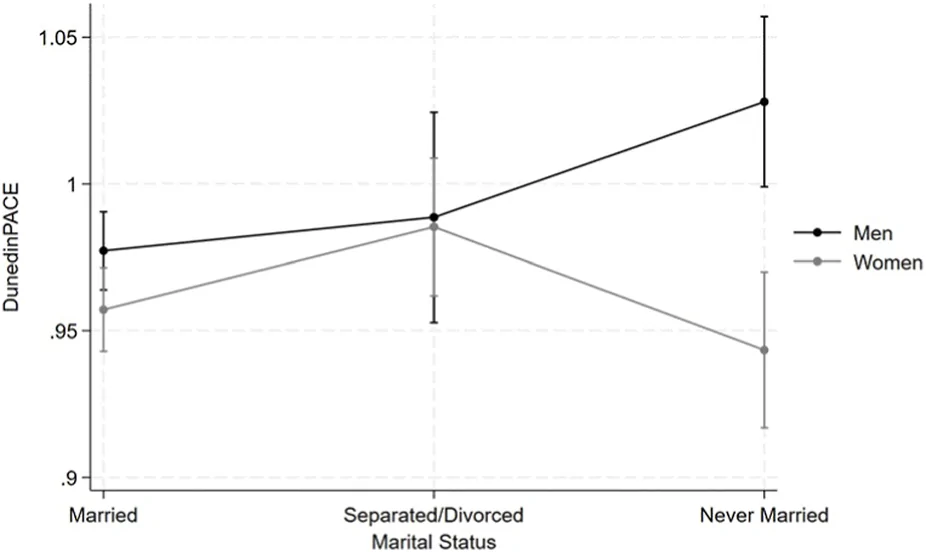
Marital status x gender interaction in relation to DunedinPACE.

The purpose of Model 2 was to investigate whether associations between marital status and epigenetic scores differs between men and women, and as such, included a multiplicative interaction term between gender and marital status. For both GrimAge and DunedinPACE, these interactions were significant and suggested that never marrying was related to significantly accelerated epigenetic scores compared to married individuals (GrimAge: *b =* −2.06*, SE =* 0.84, *p =* 0.01; DunedinPACE: *b =* −0.06*, SE =* 0.02, *p =* 0.003), but this was only observed among men and not women (see Figures 1, 2).

### 3.3 Exploratory analyses

Although the focus of this study was on associations between gender, marital status, and their interaction and epigenetic age as assessed on a series of second-generation clocks, the wide age range of the MIDUS sample provided an opportunity to investigate potential age differences in the benefits of marriage on epigenetic age. As such, a series of weighted linear regressions were conducted for each epigenetic clock that included a multiplicative interaction term between chronological age and marital status, adjusting for all covariates. There were no significant interactions between chronological age and marital status in association with any of the evaluated epigenetic clocks.

## 4 Discussion

A large body of research has indicated health benefits associated with being married, but that this circumstance is sometimes more beneficial for men than women (Monin and Clark, 2011; Wang Y. et al., 2020). The current study extended this body of research to epigenetic aging outcomes, an objectively assessed, early biological risk marker for premature aging, morbidity, and mortality in a U.S. sample of adults representing a wide age range. Some research has already documented the benefits of marriage regarding epigenetic age, with married individuals aging relatively slower than their non-married counterparts (Prigerson et al., 2025; Rentscher et al., 2023). One possible explanation for this finding may lie in the companionship one gains from marriage, as others have observed slower epigenetic aging among individuals who report lower loneliness (Freilich et al., 2024). Other existing work, however, has demonstrated that associations between marital status and epigenetic age depend, in part, on the epigenetic clock assessed (e.g., PhenoAge, GrimAge, DunedinPACE, Zhang) and the specific marital status (e.g., never married, divorced, widowed, remarried) being compared (Yu, 2023). In the present study, our initial models yielded a similar pattern on DunedinPACE; married participants had aged at a decelerated rate relative to participants who had been separated or divorced. The novel finding in the present study, however, was a gender difference specific to never marrying, a marital status that carried epigenetic risk, but only for men in this sample.

### 4.1 The never married: A greater risk for men

Previous studies have found that being married more strongly associates with better cardiovascular health and reduced cancer and mortality risk among men than women (Monin and Clark, 2011; Wang Y. et al., 2020). Findings such as these might suggest that the health benefits associated with marriage are more notable for men than women. More recent research has found that marriage, as compared to being never married, separated, or widowed, is associated with decelerated epigenetic aging (Rentscher et al., 2023). Considering the connection between accelerated epigenetic aging and health (Faul et al., 2023), an important next step has been under investigated, namely, the potential for gender differences in associations between marital status and epigenetic aging.

The present study took a nuanced view of this question by comparing men and women for associations between multiple marital statuses and rate of epigenetic age/aging on two clocks and one algorithm. This approach enabled tests not just of gender differences in the benefits of marriage on epigenetic outcomes, but of which non-married status carries greater epigenetic risk for men and women. Results revealed a divergence between men and women in a specific marital circumstance: never marrying. A recent study investigating GrimAge among married and unmarried older adults similarly observed that never marrying was the marital category related to accelerated GrimAge among men, whereas becoming widowed was the category related to accelerated GrimAge among women (Cui et al., 2025). Although the current sample was not sufficiently powered to include widowed individuals, results of the present study aligned and provided a proper statistical test to further support this unique risk for men.

One reason for this divergence may lie in the structure of roles carried out by men and women in marriages. Researchers have long posited that women experience role burden or conflicts to a greater extent than do men, or that women take on more domestic responsibilities than do men even should they also work outside the home. In her seminal work *The Second Shift: Working Families and the Revolution at Home* (Hochschild and Machung, 1989), Hochschild wrote “One reason that half the lawyers, doctors, businesspeople are not women is because men do not share the raising of their children and the caring of their homes.” And this caregiving burden is not limited to raising children. For decades, researchers have documented the mortality risk associated with caregiving for a spouse with a disability or poor health (Schultz et al., 2017), and this caregiving burden is far more likely to fall upon women (Pinquart and Sörensen, 2006). More recent research investigated the association between providing care for grandchildren and accelerated epigenetic age/ing and found this association only among grandfathers but not grandmothers (Bhat et al., 2026). However, the association between grandchild caregiving and accelerated epigenetic age was attenuated among grandfathers who reported more support from their spouses, again demonstrating the benefits of marriage for men, particularly when they are in supportive marriages. The work from Bhat et al. (2026) just described and our own may be reflective of findings suggesting that, in the presence of psychological wellbeing, psychological stress is less likely to associate with accelerated epigenetic age (Cha et al., 2025). Having a supportive marital partner likely contributes to psychological wellbeing, and not having a supportive partner may be particularly risky for men.

Furthermore, women are often more negatively impacted by marital conflict than are men (Wanic and Kulik, 2011). Women report being more distressed under hostility and exhibit lower physiological and psychological health than their partners when they are in unsatisfying marriages (Kiecolt-Glaser and Newton, 2001; Levenson et al., 1993; Noller and Fitzpatrick, 1990). Marital conflict may be more impactful for women should they hold more relationally dependent self-representations, so conflict in relationships could negatively impact the sense of self (Kiecolt-Glaser and Newton, 2001). Alternatively, the subordination reactivity hypothesis offers an additional perspective suggesting that women are further impacted by conflict due to occupying positions of less power relative to their partners (Wanic and Kulik, 2011). A subordinate position leads to less autonomy and access to material resources which can impact the power dynamic in a marriage; a man may be able to walk away from conflict and retain autonomy, but women may be relatively more dependent on the marriage. Together, these theories support the idea that marriage may introduce more opportunities for chronic strain among women which may have been less characteristic of the lives of women who had never married in this sample. This possibility is important to consider in light of research demonstrating links between higher circulating levels of the stress-related hormone, cortisol, and accelerated epigenetic aging (Takeshita et al., 2025). Should chronic strain result from some marriages, this strain may have implications for hormonal risk in the rate of epigenetic aging. Although one recent study found no such association between marital risk or partner strain on rate of epigenetic aging, that study did not report on potential gender differences in their null main effect, leaving the possibility that the strain-accelerated epigenetic aging link may have been present among women (Rodrigues et al., 2025). We view the current findings as a first step in a larger framework in which gender and marital characteristics might be evaluated for their interactive associations with epigenetic outcomes.

### 4.2 Why not PhenoAge?

Associations between marital status and epigenetic age are weaker for PhenoAge. Yu (2023) compared married individuals to several categories of unmarried individuals using the same three clocks/algorithms as in the present analyses. In Yu (2023), married women were epigenetically younger than all other women on GrimAge and DunedinPACE, but not PhenoAge. Among men, however, being married was associated with decelerated epigenetic age when compared to all other men on all three epigenetic outcomes, yet differences were most consistent on the GrimAge clock (patterns differed across models with different sets of covariates). Results from the current study were somewhat similar, namely, because being married was related to younger GrimAge and to a lesser degree DunedinPACE, although not PhenoAge. These findings also align with the work of others who have reported that individuals with greater connections to network members across religious, community, and family (parent-child) domains exhibit slower/younger epigenetic age, but more so for second-generation clocks and algorithms like GrimAge and DunedinPace than many other versions of epigenetic age assessments (Ong et al., 2025). That said, far more research is needed to investigate the robustness of these associations.

Variability in the strength of marital status-epigenetic associations may also be attributed to the construction of the different epigenetic outcomes. GrimAge was constructed using seven DNAm surrogates for a set of plasma proteins and smoking pack-years that predict mortality risk (Lu et al., 2019). PhenoAge, on the other hand, was constructed using DNAm surrogates for biological indicators of health and aging (Levine et al., 2018). It remains a possibility that some of these biomarkers are more sensitive to the benefits and/or challenges unique to marital status. Relatedly, others have found that different forms of stress, comparing perceived stress to stress related to life events, differentially associated with aging assessed on PhenoAge, GrimAge, and DunedinPACE (Goering et al., 2026). Although experiencing life events such as loss of family members through death or divorce, exposure to victimization or natural disasters, and other acute events associated with accelerated aging on all three of these clocks and algorithms, only perceived stress associated with accelerated GrimAge. Considering that finding in relation to our own, it is plausible that never marrying is an ongoing circumstance contributing to greater perceived stress among some men. Although this possibility remains speculative, it also represents an important step for future research, particularly in data sets with richer data indicating the reason for never marrying.

Moreover, researchers have argued that one mechanism leading to better health among married individuals is the social control of health behaviors (e.g., smoking; Robles, 2014). There is a well-known mortality risk associated with smoking. Perhaps the greater benefits of marriage in relation to GrimAge among men in the present study can be explained by such social control, although the potential for marital status, smoking, and epigenetic changes lying on the same causal pathway remains, to date, only speculative. Nevertheless, as an *ad hoc* exploration of the possibility that residual confounding (beyond that which was covaried by including smoking as an additional predictor in our analytic models), we examined rates of smoking across men in different marital categories. A slightly larger percentage of married men had never smoked (59 percent) or had quit smoking (35 percent), and a smaller percentage of married men were current smokers (five percent) relative to unmarried men (these percentages were 58, 26, and 16 among unmarried men, respectively). Married men in this sample, then, may have benefitted from social control of smoking behaviors. More work is needed in this area to further inform mechanisms.

### 4.3 Marriage and epigenetic clocks: Does life stage make a difference?

In our comparison of married individuals to either separated/divorced or never married individuals, we observed significant gender differences on GrimAge and DunedinPACE. This contrasted with another study of exclusively older adults which reported no gender differences (Yu, 2023). Although formal statistical tests of gender differences were not conducted in that published report, evidence generally suggested decelerated epigenetic age among married individuals compared to all other marital statuses with no notable gender differences (Yu, 2023). Although one plausible reason that Yu (2023) did not observe gender differences may have been that marital effects on epigenetic age were stratified by gender rather than (multiplicative) interacted with gender, another may have been the age distribution with which these associations were evaluated. The MIDUS II and Refresher samples examined in the current study, though representing a wide age range, was not restricted to older adulthood. Although exploratory analyses in the current study revealed no significant age differences in the associations between marital status and epigenetic age, the disparate findings between the current study and that of Yu (2023) may indicate unique features of midlife that further differentiate marriage-epigenetic associations by gender at this stage of life.

Ours are not the only results that have implied there could be greater gender differences in marital status-epigenetic associations in midlife. Wang W. et al. (2023) utilized a national United Kingdom sample representing a wide age range; although being widowed or divorced related to accelerated epigenetic scores among men and women when considering the full age range of their sample, there were some gender differences at earlier life stages. Specifically, younger single men had younger PhenoAge and decelerated DunedinPACE when compared to their same-aged married counterparts, but the opposite was true for older men. Among women, there were no significant differences in PhenoAge or DunedinPACE, either by marital status or age. Although Wang W. et al. (2023) did not evaluate GrimAge in their study, their results align with those reported in the current study in the sense that the benefits of marriage may diverge more for men and women before older adulthood is reached.

One explanation for potential life stage differences in marital status-epigenetic age associations may lie in underlying health status. At older ages, it becomes increasingly likely that both men and women have acquired at least some health problems. Deteriorating health status may, in turn, shift the costs and benefits of marriage for both men and women. For instance, marriage may be beneficial due to pooled financial resources, increased emotional and social support, and positive coercion towards healthy activities (Liu and Umberson, 2008). These factors may become equally important for men and women as one’s health deteriorates. Relatedly, others have argued that a lack of gender differences in the marital status-epigenetic association at older ages may be explained by selection effects, or that men who survive to older ages had more protective and fewer risk factors for poor health over the life course (Yu, 2023). Several other studies have observed that gender differences in associations between marital status and health outcomes attenuate among older samples of adults (Staehelin et al., 2012; Tuoyire and Ayetey, 2018; Williams and Umberson, 2004), and our findings may further corroborate this possibility. Evaluating the current research questions using samples from more recent cohorts of midlife adults is needed to determine the robustness of our findings, particularly as gender norms shift.

### 4.4 Limitations and future directions

Although this study generated important new knowledge regarding gender differences in marriage and health literature, it was not free of limitations. For instance, although investigating marital status-epigenetic associations using the MIDUS II and Refresher is a unique strength of the present contributions, this sample with data on the epigenetic clocks is small, precluding the ability to perform important sensitivity analyses related to the racial/ethnic or age composition of the sample, for example,. Furthermore, MIDUS staff utilized single blood samples to derive epigenetic scores among participants, limiting our analysis to a cross-sectional design. Future research would benefit from the use of several DNAm samples taken over multiple waves which would allow for the investigation of marriage-epigenetic associations over time. Longitudinal studies may reveal how health and epigenetic aging vary depending on the length of marriage or transitions into different marital statues (Siegler et al., 2013) which may, in turn, vary by gender (Robards et al., 2012). Additionally, although the identification of gender differences in the marriage-epigenetic associations in the present study enhances understanding of gender-related health disparities, it is presently unknown *how* marriage influences epigenetic aging. Future research should investigate potential mechanisms that could serve as worthy targets for interventions to enhance the benefits of marriage. For instance, recent research has reiterated that epigenetic change is only one factor associated with aging (Markon et al., 2025). Other aging factors including cognitive function such as memory and executive function not only relate to rate of epigenetic aging, but also more strongly predict mortality than epigenetic aging (Markon et al., 2025). Although we are far from understanding which aging process contributes most to marital status-health and mortality associations, this work invites further examination of whether marriage offers stimulation or boosts to cognitive function that, in turn, decelerates the rate of epigenetic aging. Alternatively, marriage may provide more social control over other health behaviors such as physical activity or alcohol consumption not investigated in the current study. Moreover, these modifiable health behaviors may initiate or exacerbate common pathophysiology underlying the very cardiovascular diseases that result from accelerated epigenetic aging. Although we investigated associations between BMI and epigenetic outcomes in the current study, other candidate biomarkers worthy of future study include glucose metabolism and kidney function, for instance. The present findings were observed in a sample that was primarily non-Hispanic White, and the results of this study may not generalize to a wider array of racial and ethnic groups that are nonetheless large segments of the U.S. population. For example, despite higher marriage rates among non-Hispanic White compared to non-Hispanic Black adults, some research has demonstrated a greater benefit of marriage for the longevity of non-Hispanic White relative to non-Hispanic Black adults, while other research failed to find significant racial differences in the association between marital status and measures of physiological wellbeing (Assari and Bazargan, 2019; Tobin, Robinson and Stanifer, 2019). Some research has demonstrated that non-Hispanic Black men who were married exhibited younger GrimAge, and Hispanic men who were married had slower DunedinPACE, yet non-Hispanic White women who were married had older Hannum age (Shen et al., 2025). The current sample was limited in size, precluding the ability to make these racial/ethnic comparisons. These findings nevertheless call for more research using diverse samples. Lastly, widows were excluded from the current analysis due to the small size of this group, and MIDUS II does not ask participants to report whether they are in same- or different-sex couples. Results of the current study may not generalize across these different relationships.

## 5 Conclusion

This study made important extensions to the literature describing greater risks associated with never marrying among men. The consequences associated with accelerated epigenetic aging necessitates research identifying features of marriage that carry risk for some people. The present research also motivates further identification of resources for those who do not marry, particularly for men. Although the current findings are a first step in identifying gender differences, they also encourage far more research into mechanisms for such differences. Intervention efforts may include marital therapy increasing support and decreasing strain in marital unions, or providing couples with needed skills to conjointly increase health-enhancing (e.g., diet and exercise) and decrease health-compromising (e.g., smoking) behaviors.

## Data Availability

Publicly available datasets were analyzed in this study. This data can be found here: The publicly available data used in this report are accessible at the Inter-University Consortium for Political and Social Research, https://www.icpsr.umich.edu/sites/icpsr/search/studies?q&equals;Midlife+in+the+United+States.
