# Supplementary material for: Gender differences in the association between marriage and epigenetic age
Source: Front Aging. 2026 Sep 7;7:1931190. doi: 10.3389/fragi.2026.1931190 (PMC13590688; doi:10.3389/fragi.2026.1931190)
Supplement: Supplementary file 1 [file Table1.docx]

| Supplemental Table 1 | | | | |
| --- | --- | --- | --- | --- |
| *Weighted Linear Regressions Predicting Two First-Generation Epigenetic Outcomes, Midlife in the United States Survey, MIDUS II and Refresher Samples, n = 738* | | | | |
|  | Hannum | | Horvath | |
|  | Model 1 | Model 2 | Model 1 | Model 2 |
| Married |  |  |  |  |
| Separated/Divorced | 0.65 (0.41) | 0.07 (0.69) | 1.28^**^(0.46) | 1.30 (0.78) |
| Never Married | -0.48 (0.42) | -0.36 (0.58) | 0.61 (0.47) | 0.40 (0.66) |
| Women^a^ | -1.43 ^***^  (0.29) | -1.51 ^***^ (0.35) | -1.00^**^ (0.33) | -1.07^**^ (0.39) |
| Married x Gender^a^ |  |  |  |  |
| Separated/Divorced |  | 0.84 (0.84) |  | 0.00 (0.95) |
| Never Married |  | -0.21 (0.78) |  | 0.39  (0.88) |
| Age (in years) | 0.83^***^ (0.01) | 0.83^***^ (0.01) | 0.81^***^ (0.01) | 0.81^***^ (0.01) |
| Four-year degree or higher^b^ | 0.41 (0.30) | 0.40 (0.31) | -0.11 (0.34) | -0.11 (0.34) |
| Non-Hispanic White^c^ | 0.56 (0.38) | 0.56 (0.38) | 0.10 (0.43) | 0.11 (0.43) |
| Refresher^d^ | 0.02 (0.59) | 0.05 (0.59) | 1.11 (0.66) | 1.09 (0.67) |
| Body mass index | 0.06^**^ (0.02) | 0.06^**^ (0.02) | 0.03 (0.02) | 0.03 (0.02) |
| Never Smokers |  |  |  |  |
| Smokers who had quit | 0.16 (0.34) | 0.19 (0.34) | 0.50 (0.38) | 0.48 (0.39) |
| Current smokers | 0.38 (0.45) | 0.42 (0.46) | 0.05 (0.51) | 0.02 (0.52) |
| Number of months between projects | 0.09^***^ (0.02) | 0.09^***^ (0.02) | 0.10^***^  (0.02) | 0.10 ^***^ (0.02) |

*Note*. All coefficients are *b* (*SE*)

^a^ Compared to men; ^b^ Compared to two-year degree or less; ^c^ Compared to non-White participants;

^d^ Compared to MIDUS II Core Sample

^*^ *p* < .05; ^**^ *p* < .01; ^***^ *p* < .001
